# Fourteen (14) months follow up of traumatic sciatic neuritis due to intramuscular injection: a case report

**DOI:** 10.11604/pamj.2021.39.188.29223

**Published:** 2021-07-08

**Authors:** Ashish Ramesh Varma, Arjun Rajesh Jaiswal, Sushma Balkrishna Myadam, Anubhuti Sunil Dixit, Anuj Ramesh Varma, Sakshi Pritam Arora

**Affiliations:** 1 Department of Pediatrics, Jawaharlal Nehru Medical College, Datta Meghe Institute of Medical Sciences, Wardha, India,; 2 Jawaharlal Nehru Medical College, Datta Meghe Institute of Medical Sciences, Wardha, India,; 3 Department of Medicine, Jawaharlal Nehru Medical College, Datta Meghe Institute of Medical Sciences, Wardha, India,; 4 Community Health Physiotherapy, Ravi Nair Physiotherapy College, Datta Meghe Institute of Medical Sciences, Wardha, India

**Keywords:** Intramuscular, injection, traumatic, sciatic nerve injury, physiotherapy, case report

## Abstract

The injury caused due to the intramuscular (IM) mode of drug administration are still affecting population in rural area more than urban area. The IM injection given in any quadrant except the upper outer quadrant of buttock most commonly damages the sciatic nerve because of its course and extent in the injection prone site. The iatrogenic sciatic nerve injury because of IM injection in dorsogluteal site is a matter of concern all over the world covering the undeveloped, developing and developed countries. The iatrogenic sciatic neuritis causes severe neurological or motor deficits leading to the medico-legal consequences. An 8-year-old male child, post dorsogluteal IM injection for mild fever and cold, presented left lower limb weakness and pain in left gluteal region. The patient underwent the medical and physiotherapeutic management for 14 months. The medical management included the initial dose of steroids and ox carbamazepine along with methylcobalamine and folic acid. The physiotherapeutic intervention concentrated on the functional independency of child. The patient attended complete physiological and functional recovery by the end of 14^th^ month concluding that sometimes waiting for lesion to resolve is better than intervention. The iatrogenic sciatic neuritis is a complication that needs attention for prevention following intramuscular drug administration technique.

## Introduction

Intramuscular (IM) injection is considered as one of the important mode of drug administration. The site of drug administration as well as the drug administered play an important role in recognising the injection induced complications. One such complication of IM injection is iatrogenic injury to the nerve. The sciatic nerve follows a course which passes through the gluteus muscle. The ideal site of IM injection in the buttock is the upper outer quadrant of buttock [1]. The IM injection if given in any other quadrant other than the upper outer quadrant then it may cause damage to the sciatic nerve [2]. The incidence of iatrogenic sciatic nerve injury has reduced along the decade but still is a matter of concern affecting more of rural than urban population [3]. The iatrogenic sciatic neuritis is observed as one of the persistent iatrogenic complication of IM injection all over the world healthcare systems. The patient presenting the injection induced neuritis may present the symptoms ranging from paresis to complete paralysis, mild to severe pain, sensory loss and motor involvement along with poor prognosis.

The extent of damage to the nerve due to IM injection depends on both the site of injection and the injected drug. The pathophysiology of injury involve the damage to the nerve fibres that may have occurred either because of the needle or because of the chemical composition of drug administered [4]. Also, the dosage of administered drug plays an important role in an intrafasicular injection. The iatrogenic injuries present poor prognosis. The patients presenting the symptoms typically presents the immediate onset of paresis and pain following the nerve course, with the numbness gradually following the complete sensory loss. The onset and presentation of symptoms immediately after an IM injection considering the severity of damage to the nerve needs an extensive keen medical management followed by the physiotherapeutic advancement for the complete recovery. However, the body follows an auto-repair mechanism which allows the lesion caused to the nerve to repair itself during the course of recovery.

One such unique case is reported of an 8-year-old male child presenting with left lower limb weakness and pain in left gluteal region caused immediately after dorsogluteal IM injection for mild fever and cold [2]. The patient initially underwent the medical management that included the dose of steroids and ox carbamazepine along with methylcobalamine and folic acid. The physiotherapy management concentrated on the muscle power using functional electric stimulation (FES) [5] of nerve and tone improvement using the proprioceptive neuromuscular facilitation (PNF) [6].

## Patient and observation

**Presentation of case**: a unique report of iatrogenic sciatic neuritis was reported in an 8-year-old male child as an iatrogenic complication of IM injection. The patient was manged conservatively with initial dosage of medication and prolonged physiotherapy. The presented symptoms included weakness of left lower limb and pain in left gluteal region.

**Patient**: an 8-year-old male child came to paediatric outpatient department (OPD) with chief complaints of weakness of left lower limb from 2 month and pain in left gluteal region. Patient was apparently alright 2 month back when he developed mild fever with cold for which he sought opinion from local physician who administered some medication via intramuscular route to left gluteal region. He felt intense pain after injection and started to feel sudden onset of weakness in left lower limb. The patient was weighing 18kg with 110cm height concluding his body mass index (BMI) to be 14.9 making him fall in the underweight category. On observation, patient presented an externally rotated and abducted left lower limb with no such presentation on right side.

**Clinical Findings**: on motor examination the tone of right lower limb was normal however that of left lower limb was illustrating hypotonia. The findings included the reflexes which were normal in upper limb with positive Babinski on left side as shown in Table 1. The power was intact for bilateral upper limb but showing alterations in lower limb as shown in Table 2. The calves of left side were atrophied and plantar-flexors were weak. The patient was given methylcobalamine, folic acid, ox carbamazepine and steroids.

**Table 1:** deep tendon reflexes

Deep tendon reflexes	Biceps jerk	Triceps jerk	Supinator jerk	Knee jerk	Ankle jerk
Right	+	+	+	+	+
Left	+	+	+	+	Absent

**Table 2:** power in the lower limb

Right	Power	Left
	**Hip**	
5	Flexors	4
5	Extensors	4
5	Abductors	4
5	Adductors	4
5	Internal rotators	4
5	External rotators	4
	**Knee**	
5	Flexors	4
5	Extensors	4
	**Ankle**	
5	Dorsiflexors	4
5	Plantarflexors	4

**Timeline**: historical and current information from this episode of care organized as a timeline (Table 3).

**Table 3:** the sequence wise duration of 14 months follow-up treatment program from date of assessment to follow-up

Initiation of episode	02/11/2019
Date of Admission	04/02/2020
Date of Assessment in Medicine OPD	04/02/2020
Date of Assessment in Physiotherapy OPD	04/03/2020
Follow-up date	04/04/2021

**Figure 1:**
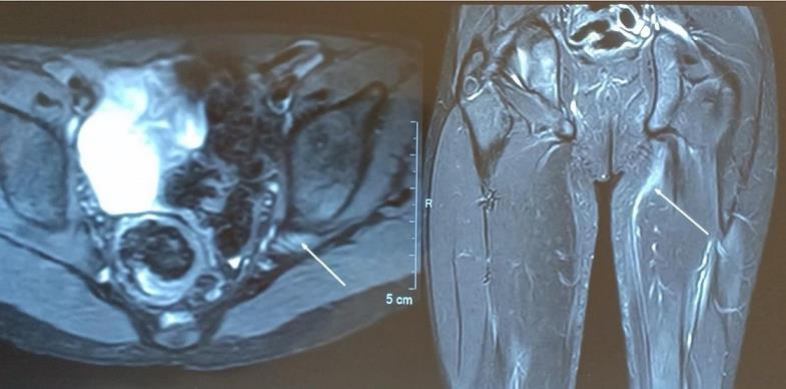
magnetic resonance imaging locating the peripheral nerve lesion

**Figure 2:**
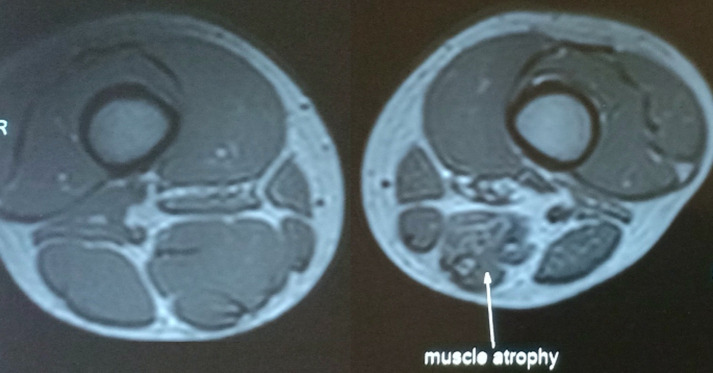
magnetic resonance imaging showing muscle atrophy

**Diagnostic assessment**: assuming this as a case of traumatic sciatic nerve injury the magnetic resonance imaging (MRI) as shown in Figure 1 and Figure 2, was done which showed flattening and oedema involving left sciatic nerve at level of greater sciatic foramina, hip joint level extending just proximal to ischeal tuberosity. The signs of possibility of sciatic nerve injury was found with the continuity of nerve being relatively maintained on MRI stating predominant involvement was for length of 5-6 cm. Also, the rest of the sciatic nerve was normal in thickness except for with only subtle hyperintensity along with denervation atrophy of the hamstring muscle along mid and lower thigh. The nerve conduction study (NCV) stated that the compound muscle action potential (CMAP) amplitude could not be elicited in right tibial nerve. Also, in peroneal nerve the CMAP amplitude was reduced with normal motor latency and conduction velocity. However, the sensory nerve action potential (SNAP) amplitude and conduction velocity was within normal limits in bilateral sural nerve with more focus on left sciatic nerve involvement. Again, the NCV was conducted after 5 months demonstrated axonal lesion of left sciatic nerve affecting tibial division predominantly with no sign of reinnervation in tibial division.

**Therapeutic intervention**: patient was started on initial dose of steroids and ox carbamazepine along with methylcobalamine and folic acid and was referred to physiotherapy. The regular physiotherapy included group muscle stimulation of the sciatic nerve motor point for 20 minutes with an interval of 10 seconds after every 30 set of contractions. The proprioceptive neuromuscular facilitation (PNF) was added for improving the tone and strengthening of the respective muscles. The frequency of the treatment was 45 minutes session daily with 6 days a week for 14 months. With regular follow up and physiotherapy patient had regained power 5/5 in left lower limb and the left calf muscle also regained normal size. The normal functional independency of the patient was observed by the end 14 months from the initiation of treatment with improvement of power in each muscle group of lower limb.

**Follow-up and outcomes**: the lower extremity functional scale (LEFS) was used to assess patient´s initial function, ongoing progress, and outcome in terms of functional independence. The results concluded the patient´s status from “extremely difficult to perform activity”, to “no difficulty while performing same activity” [7]. While assessing the patient´s ability in terms of independence, no adverse and unanticipated events were observed.

## Discussion

The administration of drugs through the IM injection may cause damage to the peripheral nerve either by piercing the sharp tip of needle in the nerve fibres or by administering the pharmacological compound into the nerve fibres damaging its normal anatomical structure and physiological function [8]. The high potential of iatrogenic sciatic neuritis is considered significant because of association of preferred choice of administration of IM injection and location of sciatic nerve. The magnetic resonance imaging [9,10] and electrodiagnostic studies involving electromyography and nerve conduction velocity [11] played an important role in following up the recovery status while lesion was healing itself with auto-repair mechanism. Also, it assisted in initially locating the injury, grading the extent of nerve damage and following the prognosis.

The physiotherapeutic functional electrical stimulation (FES) is used to prevent muscle atrophy and to preserve and improve muscle strength [5]. The application of PNF for improvement of muscle tone concentrated on the muscle activation and output of power leading to functional improvement [6]. In the current reported case, the left lower limb weakness and left gluteal pain due to sciatic nerve injury was remarkable and early medication with prolonged physiotherapy was undertaken. The lesion was given time for auto-regeneration without superseding it with intervention in order to prevent any other associated complication.

**Patient perspective**: the patient shared their perspective on the treatment in terms of functional independence stating the improvement in the activity limitation and participation restriction of the patient.

**Informed consent**: the patient had given written informed consent.

## Conclusion

A paediatric patient with iatrogenic sciatic neuritis caused immediately post misplaced IM injection presenting neurological sequences is reported. The patient attended complete physiological and functional recovery by the end of 14^th^ month concluding that sometimes waiting for lesion to resolve is better than intervention. However, the health professionals must attentively administer the IM injection in order to reduce the incidence of iatrogenic nerve injury.
